# Hyperthyroidism and Wnt Signaling Pathway: Influence on Bone Remodeling

**DOI:** 10.3390/metabo13020241

**Published:** 2023-02-06

**Authors:** Dunja Mudri, Ines Bilić Ćurčić, Lucija Meštrović, Ivica Mihaljević, Tomislav Kizivat

**Affiliations:** 1 Department of Nuclear Medicine and Oncology, Faculty of Medicine, University of Osijek, 31000 Osijek, Croatia; 2 Clinical Institute of Nuclear Medicine and Radiation Protection, University Hospital Osijek, 31000 Osijek, Croatia; 3 Department of Pharmacology, Faculty of Medicine, University of Osijek, 31000 Osijek, Croatia; 4 Department of Endocrinology and Metabolism Disorders, University Hospital Osijek, 31000 Osijek, Croatia; 5 Faculty of Medicine, University of Osijek, 31000 Osijek, Croatia; 6 Academy of Medical Sciences of Croatia, 31000 Osijek, Croatia

**Keywords:** hyperthyroidism, bone density, Wnt inhibitors, sclerostin, DKK1, thyroid

## Abstract

Graves’ disease is an autoimmune disease of the thyroid gland, characterized by increased production of thyroid hormones, which can affect many different organ systems in the body. Among other problems, it can cause disorders of the skeletal system, shortening the bone remodeling cycle and causing a decrease in bone density. The Wnt cascade signaling pathway and the β-catenin, as a part of the canonical Wnt pathway, also play roles in maintaining bone mass. Inhibition of the Wnt pathway can cause bone loss, and its stimulation can increase it. The Wnt signaling pathway influences the effectiveness of thyroid hormones by affecting receptors for thyroid hormones and deiodinase, while thyroid hormones can change levels of β-catenin within the cell cytoplasm. This indicates that the Wnt pathway and thyroid hormone levels, including hyperthyroidism, are linked and may act together to change bone density. In this review article, we attempt to explain the interplay between thyroid hormones and the Wnt pathway on bone density, with a focus on directions for further research and treatment options.

## 1. Introduction

Graves’ disease (GD) is an autoimmune disease of the thyroid gland in which antibodies bind to the TSH receptor in thyroid follicular cells, causing hyperplasia, increased production of thyroid hormones tetraiodothyronine (thyroxine, T4), triiodothyronine (T3), and hyperthyroidism [1]. T4 and T3 are released from the thyroid follicular cells into the circulation, mostly bound to plasma proteins, while a small fraction circulates freely.

Hyperthyroidism causes accelerated metabolism and can affect different organs and organ systems, so the symptoms and signs of the disease can be different, encompassing, among other problems, disorders of the skeletal system, shortening the bone remodeling cycle by almost 50% [2]. There is a disturbance in the relationship between bone formation and resorption, the second overcoming the first, leading to a decrease in bone density, i.e., osteopenia or osteoporosis [3,4].

Due to the increasing prevalence of osteoporosis, one of the major causes of fractures and disability in the aging population, factors important for maintaining the bone remodeling process have been systematically investigated. The Wnt cascade signaling pathway and its inhibitors play an important role in maintaining bone homeostasis [5,6,7,8]. One of the key players is β-catenin, a part of the canonical Wnt pathway. Stimulation of the Wnt pathway increases bone mass, whereas its inhibition causes bone loss [9]. Therefore, inhibitors of the canonical Wnt pathway have become a focus of research, especially sclerostin and dickkopf 1 (DKK1).

In this review article, we aimed to elucidate the association between thyroid hormones and the Wnt pathway on bone density. Given the complexity of engaged interactions in the bone remodeling process, we intended to provide guidelines for further research, considering the importance of osteoporosis and hypothyroidism treatment.

## 2. Graves’ Disease

Graves’ disease is the most common cause of hyperthyroidism, in which thyroid hormones are increasingly secreted into the circulation and affect target organs. The incidence of GD is 20–50 cases per 100,000 people per year, affecting mostly adults between 30 and 60 years of age [2,10]. Women are affected 5–10 times more often than men, and the risk of developing the disease during a lifetime in women is 3% and only 0.5% in men [10]. The factors that lead to the onset of the disease are mostly genetic, in 79% of cases, while environmental factors account for 21% [2]. Typical risk factors are smoking, sex hormones, pregnancy, stress, inflammation, and inappropriate iodine intake [11].

Thyroid hormones are important for a variety of cellular functions, including proliferation, growth, differentiation, metabolism, regeneration, and homeostasis [12].

**Figure 1:**
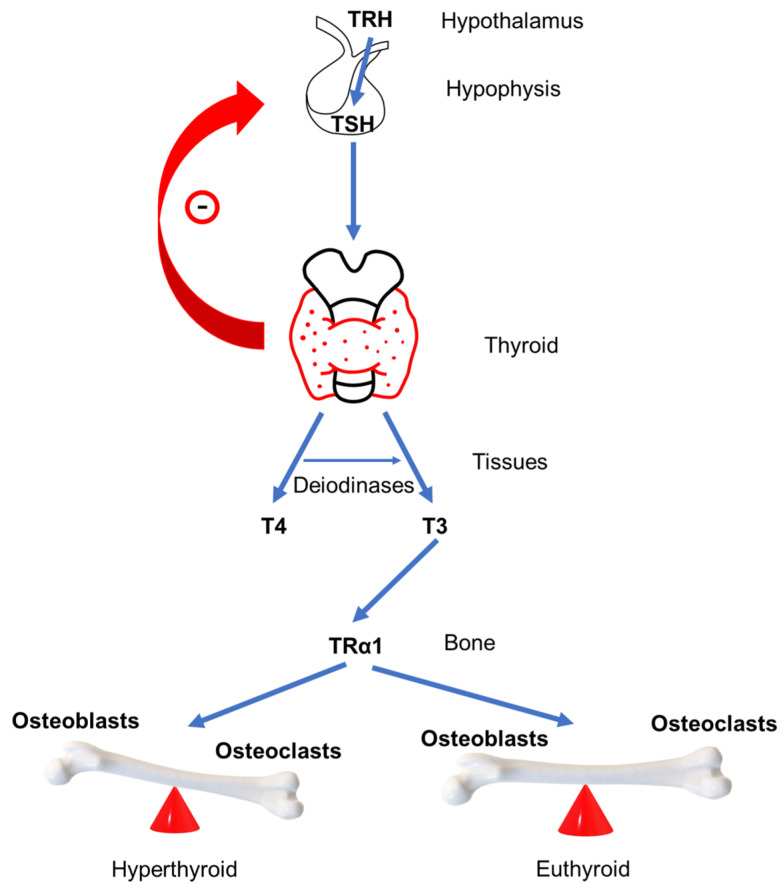
Schematic representation of the hypothalamus–pituitary–thyroid axis and effect on bone in hyperthyroidism and euthyroidism, TRH thyroid releasing hormone, TSH thyroid stimulating hormone, T4 thyroxine, T3 three iodothyronine, and TRα1 thyroid hormone receptor α.

The production and secretion of thyroid hormones is regulated by the negative feedback loop of the hypothalamus–pituitary–thyroid axis [13]. The hypothalamus produces thyrotropin-releasing hormone (TRH), which goes to the anterior part of the pituitary gland where it binds to TRH receptors stimulating the production of thyroid-stimulating hormone (TSH) [14]. Subsequently, TSH affects the production of thyroid hormones by binding to TSH receptors (TSH-R) located on the membrane of thyroid follicular cells [15] (Figure 1).

Binding of TSH to TSH-R in follicular cells stimulates glycoprotein production thyroglobulin (Tg) and the intake of iodine from the circulation into the follicular cells via the sodium-iodide symporter (natrium-iodide symporter, NIS). In the colloid, with the mediation of the thyroid peroxidase (TPO) enzyme, iodide converts into iodine and iodine binds to tyrosyl residues on the Tg molecule. Binding of one iodine to tyrosine produces monoiodotyrosine (MIT), and binding of two iodides to tyrosine produces diiodotyrosine (DIT). The union of MIT and DIT produces thyroid hormones, triiodothyronine (T3) and tetraiodothyronine (thyroxine, T4). These fusion processes are also mediated by the TPO. The next step is the endocytosis of Tg, returning Tg from the colloid to the follicular cell, where the separation of T4 and T3 from Tg takes place, enabling excretion into circulation.

The largest part of circulating thyroid hormones, about 99%, is bound to plasma proteins, and the rest circulates as unbound, free T4 (FT4), and free T3 (FT3) [16] in order to form a reserve of thyroid hormones, preventing excretion by the urine [17]. The main hormone secreted by the thyroid gland is T4 [18]. T4 acts as a prohormone for T3, and its main role is the conversion to T3 [19] in peripheral tissues by the deiodinase enzyme [20]. There are three types of deiodinases: 1, 2, and 3 (D1, D2 and D3). D1 is the most important for the conversion of T4 to T3, although the conversion is also mediated by D2. D1 and D3 mediate the conversion of T4 into metabolically inactive reverse T3 (rT3). The conversion of rT3 into T2 hormone (which is metabolically inactive and rapidly further metabolized) is mediated by D1 and D2, while the conversion of T3 into T2 is mediated by D3 [21]. Eighty percent of T3 is produced by the conversion of T4 to T3, and the remaining 20% is excreted directly from the thyroid gland. Upon entering the target cell, T3 binds to thyroid hormone receptors (TR) located in the nucleus, which regulates gene transcription. There are several forms of the receptor: TRα1, TRα2, TRβ1, and TRβ2 [22]. Both receptors, TRα1 and TRβ1, are present in bone cells, but TRα1 is more common than TRβ1 and is an important mediator of the action of T3 in bones [23].

GD is an autoimmune disease in which antibodies related to the TSH receptor (antiTSH-R) are activated, binding to TSH-R on the follicular cells of the thyroid [11] (Figure 1) and continuously and uncontrollably stimulate the follicular cells of the thyroid, which consequently produce an excessive amount of thyroid hormones, T3 and T4.

In addition to GD, hyperthyroidism can be caused by nodular diseases, such as multinodular toxic goiter (MNTG), the presence of multiple nodules in the thyroid gland, and toxic adenoma (TA), which is characterized by the presence of one nodule of a certain size in one lobe of the thyroid gland. Unlike GD, these two diseases are not caused by autoimmune processes, whereas the increased production of thyroid hormones is usually less pronounced than in GD and occurs more often in people with heart disease.

Elevated levels of thyroid hormone in the blood can also be a consequence of thyrotoxicosis. Unlike hyperthyroidism, which is the result of increased production of thyroid hormones in the thyroid gland itself, thyrotoxicosis means only their increased level in the circulation, which is not the result of increased production in the follicular cells of the thyroid. The causes of thyrotoxicosis can be inflammatory thyroid diseases, such as subacute thyroiditis, postpartum thyroiditis, silent thyroiditis, excessive iodine intake (as a result of taking drugs or performing diagnostic tests that contain inactive iodine, as well as taking dietary supplements that contain iodine). Likewise, hormone levels can be transiently elevated in another autoimmune thyroid disease called Hashimoto’s thyroiditis, which is the most common cause of hypothyroidism.

## 3. Clinical Symptoms and Treatment of GD

Thyroid hormones act on different organs and organ systems, so the symptoms and signs of the disease can be different. The most common are palpitations, fatigue, tremors, anxiety, sleep disturbance, weight loss, heat intolerance, and excessive sweating. During the physical examination, rapid heartbeat, trembling of the extremities, and warm and sweaty skin are most often present. Additionally, as part of the extrathyroidal manifestation of GD, changes in the eyes can occur, which are known as Graves’ orbitopathy (GO) [24,25].

To clinically establish the diagnosis of GD, it is necessary to determine the levels of thyroid hormones in the blood and the levels of antibodies, most importantly antiTSH-R. There are other antibodies that can be determined, such as antibodies related to TPO (antiTPO) and to Tg (antiTg). Their levels can be elevated in GD, as it is an autoimmune disease, but the most important antibody for GD is antiTSH-R, which usually correlates well with disease activity. In addition to laboratory tests, thyroid scintigraphy and ultrasound are used to determine the size and appearance of the thyroid gland. Laboratory findings characteristic of GD are elevated serum thyroid hormone levels, either T4 and T3 or FT4 and FT3, low TSH levels, or elevated serum antiTSH-R levels.

There are three ways of treating patients with GD, namely, the use of antithyroid drugs, radioiodine therapy (RITh), and thyroid surgery [26]. Treatment options may vary by geographic region. The first choice of treatment for GD is the introduction of antithyroid drugs. Treatment methods for MNTG and TA are the same as for GD, but the first choice of treatment is the use of RITh, followed by thyroid surgery or treatment with antithyroid drugs. Thyrotoxicosis is usually treated symptomatically or in the case of a certain type of iatrogenic thyrotoxicosis with the use of corticosteroids.

Antithyroid drugs actively enter the thyroid gland, where they prevent the production of thyroid hormones by inhibiting TPO. Therefore, iodine oxidation, Tg iodination, and the coupling of MIT and DIT into thyroid hormones are reduced. Moreover, the formation of Tg and the growth of follicular cells are decreased, and conversion of T4 to T3 in peripheral tissues is impaired. Since antithyroid drugs prevent the formation of new hormones, but do not prevent the release of already formed hormones into the circulation, it takes a certain amount of time for the levels of T4 and T3 in the circulation to decrease.

According to the guidelines of the European Thyroid Association, the drugs of choice in the treatment of GD are thionamides, such as carbimazole, in a dose of 10–30 mg per day and propylthiouracil (PTU) in a dose of 100 mg every 8 hours. In addition to the action above, PTU also reduces the conversion of T4 to T3 in peripheral tissues [27].

## 4. GD Impact on Bone Remodeling

The skeletal system, among others, can be affected by GD. Bones are biologically very active, and during one’s lifetime, undergo processes of bone remodeling in order to maintain their main functions: supporting the body, protecting the internal organs, and maintaining the mineral balance of the organism [28]. The skeletal system consists of cells and an intercellular matrix.

The process of bone remodeling is active throughout the lifespan, however, the processes of bone resorption and bone formation are normally in homeostasis, maintaining bone mass [29]. A stimulus for initiating the bone remodeling process can be structural damage or systemic paracrine factors regulating body mineral balance [23]. There are four phases of bone remodeling. The first phase is the activation phase, during which osteocytes are activated and regulate osteoblasts and osteoclasts activation creating bone remodeling compartment on the bone surface where osteocyte cell apoptosis occurs and cytokines and growth factors are released. This is followed by the second phase of the bone resorption. During this phase, osteoclasts drawn by the cytokines and growth factors released in the previous phase start to resorb damaged areas. In addition, released growth factors and matrix degradation proteins attract osteoblasts, followed by a third phase called the turnover phase. Bone resorption ends, and formation of bone matrix begins. Different mononuclear cells resorb bone matrix leftovers and prepare the resorbing site for the final bone formation phase. Preosteoblasts differentiate into osteoblasts, thus producing components of the intercellular matrix and regulating the mineralization of a new matrix by excreting calcium and phosphates and degrading inhibitors of mineralization. The result of the remodeling cycle is damaged bone restoration to maintain bone strength, mineralization, and structure [20,30]. The bone resorption phase normally lasts 50 days, while the bone formation phase lasts 150 days [30], which is slightly shorter than seven months. To maintain bone integrity, bone resorption and bone formation must be coordinated in space and time. In hyperthyroidism, bone remodeling cycles occur more frequently, while the cycle itself is shortened and lasts about three to four months, which is shorter by about 50% [31] in comparison to a bone remodeling cycle of normal duration, whereby bone resorption overcomes its formation (Figure 1). When GD is treated, either with pharmacotherapy or other types of treatment, the bone remodeling cycle returns to normal length, and bone density improves [32].

The action of T3 and TSH in osteocytes has not been investigated, and it is not known whether osteocytes have transporters for thyroid hormones, deiodinases, thyroid hormone receptors, or TSH receptors. On the other hand, osteoblasts have transporters for thyroid hormones, deiodinases D2 and D3, as well as thyroid hormone receptors (predominantly TRα) and TSH receptors. Most of the studies suggest that T3 enhances osteoblast differentiation and bone formation. Data on the effect of TSH are ambiguous, therefore, are not clear whether TSH acts as a stimulator, inhibitor, or has no effect on osteoblast differentiation and function. Osteoclasts also have type-3 deiodinase, thyroid hormone receptors, and TSH receptors. However, the mechanism of T3 action on osteoclasts remains unclear, whether it acts directly on osteoclasts or indirectly via osteoblasts [20].

## 5. Wnt/β-Catenin Pathway

The first member of the wingless/integrated (Wnt) pathway was discovered in 1982 [33] and has been investigated ever since. The Wnt pathway is a complex cascade signaling pathway made of at least 19 Wnt proteins, 10 transmembrane receptors from the family of Frizzled (FZD) proteins, a few different coreceptors, different inhibitors, such as low-density lipoprotein receptor-related proteins (LRP), LRP4, and LRP5/6, disheveled proteins (DVL), axin, adenomatous polyposis coli (APC), glycogen synthase kinase 3β (GSK-3β), casein kinase 1 (CK-1), β-catenin, T-cell factor/lymphoid enhancer factor (TCF/LEF), Goucho repressor, sclerostin, and dickkopf 1.

In the last couple of decades, diseases related to rare gene mutations, leading to bone density disorders, were investigated [34]. During this research, the Wnt pathway was identified as a crucial factor in bone homeostasis, made of two main components. One component is the Wnt/β-catenin pathway, also known as a canonical pathway. The second component is the noncanonical pathway, which includes the planar cell polarity pathway (PCP) and the Wnt calcium pathway (Wnt–Ca^2+^) [9,35]. All three pathways act via binding Wnt proteins to FZD receptors [36]. The canonical Wnt/β-catenin pathway regulates morphogenesis during embryonic development and maintenance of homeostasis, as well as stem cell biology [37]. The noncanonical Wnt pathway regulates more than one cell function, including proliferation, differentiation, adhesion, polarity, motility, and migration [38]. The most important and most investigated pathway is Wnt/β-catenin pathway. Noncanonical pathways are independent of β-catenin and LRP, as opposed to the canonical pathway [39]. Activation and inhibition of the Wnt/β-catenin pathway occur through several cascade steps in which different proteins take part.

**Figure 2:**
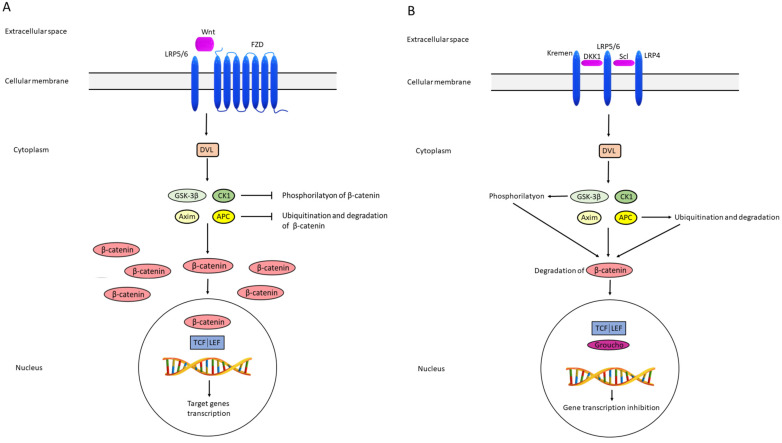
Schematic representation of activation (**A**) and inhibition (**B**) of the Wnt pathway. FZD frizzled; LRP4/5/6 lipoprotein receptor-related proteins 4, 5 and 6; DVL disheveled; GSK-3β glycogen synthase kinase 3β; CK-1 casein kinase 1; APC adenomatous polyposis coli; TCF/LEF T-cell factor/lymphoid enhancer factor; DKK1 dickkopf 1; Scl sclerostin.

Activation of the pathway begins following the binding of the extracellular cell signal to LRP5/6 and FZD receptors located in the cell membrane, whereby the extracellular cell signal usually represents one of the Wnt proteins, most often Wnt 3a and Wnt1c. FZD receptors are specific seven-pass transmembrane FZD proteins. In the cell cytoplasm, DVLs transfer the signal to a protein complex composed of GSK3β, CK1, axin, and APC. The consequence is inhibition of GSK3β and axin, leading to decreased β-catenin phosphorylation, enabling stability and accumulation of β-catenin. In the nucleus, the signal reacts with transcription factors TCF/LEF, resulting in enhanced transcription of genes relevant to cells survival, differentiation, and migration (Figure 2) [40,41].

The opposite situation is the inhibition of the Wnt signaling pathway inhibition. Factors bind to FZD and LRP5/6 in the cell membrane. This signal is passed through DVL to protein complex made of GSK3β, CK1, axin, and APC, also called the destruction complex, since GSK3β and CK1 phosphorylate β-catenin, while axin and APC cause β-catenin ubiquitination and proteasomal degradation. The consequence is the absence of interaction between β-catenin and transcription factors TCF/LEF in the nucleus. Instead of β-catenin, the Groucho repressor binds to the TCF/LEF, thus preventing the transcription of target genes [40,41] (Figure 2a,b).

The Wnt/β-catenin pathway is important for all three cell types present in the adult skeleton: osteoblasts, osteoclasts, and osteocytes, which contribute to the regulation of osteoblasts and osteoclasts during bone remodeling [42]. An activated Wnt pathway increases bone mass, while its inhibition decreases bone density [9,43]. Therefore, it is not surprising that the investigations of Wnt inhibitors, especially sclerostin and DKK, become the focus of research regarding the pathophysiology and pharmacotherapy of bone disorders.

Sclerostin and DKK1 expression is regulated by complex mechanisms, including cross-action of systemic hormones, cytokines, and mechanical load. Following mechanical stimuli, osteocytes secrete sclerostin, which travels to the bone surface, where DKK1, binds to LRP5/6. LRP4 presents sclerostin to LRP5/6 [8], while DKK1 binds to Kremen proteins [44]. These reactions prevent Wnt protein binding and inhibit signal transmission in bone cells, thereby inactivating the Wnt/β-catenin in the previously described manner. Wnt/β-catenin pathway inhibition, besides on bone cells, also has an impact on RANK/RANKL/OPG system. The receptor activator of nuclear factor kappa-B ligand (RANKL) is secreted by osteoblasts and osteocytes. RANKL binds to receptor activator of nuclear factor-kappa B (RANK), which enables activation, maturation, and survival of osteoclasts. Osteoblasts also excrete glycoprotein osteoprotegerin (OPG), which serves as a decoy for RANKL, preventing RANK to RANKL binding, leading to osteoclastogenesis inhibition. OPG is also called osteoclastogenesis inhibitory factor (OCIF), whose effects are opposite to those of RANKL. Wnt/β-catenin pathway inhibition leads to osteoblastogenesis impairment and a decrease in osteocyte survival. OPG expression is decreased in osteoblasts and osteocytes while, in osteocytes, it increases the expression of sclerostin and RANKL. These processes favor the differentiation of osteoclast precursors into osteoclasts, and the consequence is reduced formation and increased bone resorption. Additionally, increased expression of sclerostin limits bone formation through a negative feedback loop. Likewise, osteocytes’ expression and activity of the enzymes in the bone matrix are intensified, leading to increased osteolysis [45].

Sclerostin is a glycoprotein encoded by a SOST gene [46]. It is produced mainly in osteocytes [47,48] and, therefore, depends on bone mass and osteocytes’ presence. Osteocytes are embedded in the bone mineral matrix [49] and contain a wide network of canaliculi, essential for the bone remodeling process, via recognition of microfractures [50]. Sclerostin is also found in osteoblasts, chondrocytes, odontoblasts, and cementocytes, while SOST can be transcribed in bone marrow, cartilage, kidneys, liver, lungs, and pancreas [51].

In vertebrates, DKK1 is a glycoprotein and a member of a family consisting of four members (DKK1-4) [52] produced by osteocytes and osteoblasts, while its expression was also found in the skin, endothelium, prostate, placenta, platelets, and, to a lesser extent, in other tissues [53,54]. It inhibits the development and activity of osteoblasts. Therefore, increased levels of DKK1 can damage osteoblasts activity and cause bone loss [7]. Sclerostin and DKK1 antibodies are interesting as pharmacological options in osteoporosis treatment and other bone disorders [44], but sclerostin is a highly selective pathway modulator [34], possibly influencing bone formation, as well as bone resorption, thus making it a more suitable pharmacotherapeutic option.

DKK1 effects on bone mass were variable and less pronounced compared to those obtained by sclerostin antibodies administration and are achieved in two ways. One way is by causing bone erosions seen in some autoimmune diseases, such as rheumatoid arthritis, osteoarthritis, and systemic lupus erythematosus. The other way is causing osteopenia, which can be induced by glucocorticoids or estrogen deficiency [53]. In vitro studies and preclinical research provided some encouraging results on DKK1 antibody applications in orthotropic cancers [55].

Systematic investigations on inhibitors of the Wnt pathway led to the approval of a new drug for osteoporosis treatment called romosozumab [56] This drug is approved for the treatment of severe osteoporosis in postmenopausal women who have a high risk of fracture.

## 6. Wnt/β-Catenin Signaling Pathway and Thyroid Hormones

Deiodinases are, as well as TR, important for thyroid hormone action. In hyperthyroidism, the activity of D1, present in the peripheral tissue, increases. It is mostly found in the liver and kidneys and to a lesser extent in skeletal muscles, heart, and thyroid gland. The action of D1 increases the conversion T4 into T3, while D2 is responsible for the conversion of T4 into T3 inside the cell. Its activity increases in the state of thyroid gland deficiency, i.e., hypothyroidism, in order to increase the conversion of T4 into T3 [57]. Additionally, the activity of D3 is diminished to reduce the conversion of T4 to metabolically inactive forms.

The second level of action of thyroid hormones is the receptors to which they bind in the cell’s nucleus so as to pursue their activity. TRs are transcription factors that are modulated by T3. T3 has a higher binding affinity to TR than T4, which makes it a biologically more active hormone. The physiological response to T3 depends on the type of TR found in the cell. After T3 enters the cell and the nucleus, where it binds to TR, gene transcription is stimulated. T3 binds to TR, after which the corepressor is separated from TR, and the coactivator binds. This complex, TR and coactivator, binds to the promoter region of the gene and stimulates the transcription of the target genes. When there is no T3 in the cell, TR remains in complex with the corepressor resulting in the inhibition of gene transcription [58].

However, the TR function is coordinated and integrated via other signaling pathways as well, which is particularly related to the interaction between the TR pathway and the Wnt pathway. The interaction can take place in different tissues and involve either TRα or TRβ receptors. The final result of their interaction depends on the type of cells in which T3 can stimulate proliferation or differentiation, or activation or repression of the Wnt system in certain tissues [36]. In colon cancer cells, binding of T3 to TRβ1 results in suppression of the Wnt pathway, inhibiting transcription of the cyclin D1 gene in a reaction mediated by inhibition of β-catenin [59]. Additionally, the interaction between TRβ and β-catenin was investigated in mouse thyroid cancer. The results showed that TRβ binds to β-catenin, while T3 interrupted their interaction, promoting the degradation of β-catenin in the cytoplasm and, consequently, reducing the activity of the Wnt pathway [60].

The Wnt pathway can indirectly affect T3, as described in a colon cancer model. The Wnt pathway effect is mediated through deiodinases, whereby β-catenin stimulates D3, while simultaneously reducing D2. This double action results in a decrease in the intracellular level of T3 [61]. When the Wnt pathway is activated, β-catenin is not degraded in the cytoplasm, but goes to the nucleus, where it stimulates the transcription of the D3 gene. As a result, the level of D3 increases, and through its mediation, T4 is inactivated by conversion to rT3, as well as T3 to T2. It is believed that Wnt affects directly to D2, but the molecular mechanisms involved in this process have not yet been clarified [62].

Thyroid hormones are important for normal skeleton development. Several studies have investigated and demonstrated the effect of thyroid hormone on chondrocytes in growth plates by acting on the Wnt/β-catenin signaling pathway. It has been shown that thyroid hormones regulate terminal differentiation of chondrocytes in growth plates partially via the Wtn/β-catenin pathway in a study in which rat epiphyseal chondrocyte cell culture was treated with T3 hormone. The results showed an increased amount of stable β-catenin in the cytoplasm, as well as increased transcriptional activity of TCF/LEF in the nucleus, thus stimulating Runx2/cbfal gene expression, and the end result was the final differentiation of chondrocytes. Additionally, the action of DKK1 inhibited the Wnt/β-catenin pathway and, consequently, the changes induced by the action of the T3 hormone [63]. In addition, carboxypeptidase Z (CPZ) was shown to be a factor that is a part of the T3 action pathway on the Wtn/β-catenin pathway. T3 stimulated CPZ expression, and CPZ stimulated signal transmission in the Wnt pathway, leading to chondrocyte differentiation in the growth plate [64]. Another mediator of the interactions between T3 and the Wtn/β-catenin pathway is insulin-like growth factor 1 (IGF-1), which stimulates the expression of Wtn4 and acts as a β-catenin stabilizer [65]. Additionally, IGF-1 receptor expression is stimulated by T3.

There are only a few studies investigating the association between thyroid hormones, the Wnt signaling pathway, and bone. An animal model was designed mainly for testing thyroid cancer [60,66], and the experimental mice had a mutation in the gene for TRβ. In mouse osteoblasts, a dominant negative mutation of the TRβ gene led to activation of the Wnt/β-catenin pathway and increased bone formation [67]. Based on in vitro studies in which transfection induced the expression of Thrb1 and Thra1 in osteoblast cells, it was suggested that free TRs act as a stabilizer of β-catenin in bone. This effect was interrupted by the binding of T3 to TR, leading to the degradation of β-catenin and inhibition of the Wnt pathway [60,67].

Studies on the association between elevated thyroid hormone levels, the Wnt signaling pathway, and bone are lacking. In two studies involving hyperthyroid patients due to GD or MNTG conducted by the same authors, a significant decrease in serum sclerostin concentration was achieved after four to six weeks of treatment with antithyroid drugs when the euthyroid state was accomplished [68,69]. These results match other studies in which significantly elevated serum sclerostin concentrations were in the hyperthyroid state compared to euthyroid state [32,70], as well as in a study involving hyperthyroid mice [71]. However, opposite results are also recorded, since no significant difference in serum sclerostin levels was obtained between hyperthyroid patients and euthyroid controls [72]. As far as we know, there are two studies on animal models and one study conducted in humans regarding the influence of elevated thyroid hormone levels on DKK1 concentrations. Two studies investigated whether hyperthyroidism is associated with DKK1 [32,71]. In hyperthyroidic mice, serum DKK1 concentrations were decreased [71]. Similar results were shown in a study including human hyperthyroid participants with GD, comparing control serum DKK1 measurements in the euthyroid state with the hyperthyroid state, where an increase in serum DKK1 concentrations was observed [32]. Additionally, when the role of DKK1 was assessed in thyroid hormone–induced changes in bone using conditional DKK1 knockout mice, it was shown that loss of DKK1 is not sufficient to fully reverse changes in bone mass and bone turnover [73].

The results of the majority of the studies (shown in Table 1) indicate that serum concentrations of Wnt inhibitors, sclerostin, and DKK1 are changing depending on thyroid function status. Serum sclerostin levels are higher in hyperthyroid versus euthyroid state, while DKK1 levels are lower in the hyperthyroid state and increase while reaching the euthyroid state. Therefore, these results are indicative of existing cross-talk between thyroid hormones and the Wnt signaling pathway. It would be interesting to investigate changes in serum concentrations of these two inhibitors after achieving the euthyroid state given the time period in which bone density improves can vary. Consequently, sclerostin and DKK1 could serve as biomarkers in diagnosis and follow up of diseases involving the bone system.

**Table 1:** List of studies investigating hyperthyroidism and Wnt inhibitors, and this includes main findings.

Author	Study Design	Hyperthyroidism Etiology	Analyzed Wnt Inhibitor	Main Results
Tsourdi 2015 [71]	Animal models	Induced hyperthyroidism	Sclerostin, DKK1	Increased sclerostin and decreased DKK1 serum concentrations in hyperthyroid mice;
Tsourdi 2019 [73]	Animal models	Induced hyperthyroidism	DKK1	Loss of DKK1 is not sufficient to fully reverse thyroid hormone– induced changes in bone mass and bone turnover
Skowrońska-Jóźwiak 2012 [68]	In 15 patients, sclerostin was measured at diagnosis of hyperthyroidism and after 6–10 weeks of treatment with thiamazole	Graves’ disease or toxic multinodular goitre	Sclerostin	A significant decrease in serum sclerostin levels after achieving euthyroid state
Skowrońska-Jóźwiak 2015 [69]	In 33 patients, serum sclerostin was measured at diagnosis of hyperthyroidism and after 6–10 weeks of treatment with thiamazole	Graves’ disease or toxic multinodular goitre	Sclerostin	After treatment of hyperthyroidism, a significant decrease in serum sclerostin was measured.
Sarıtekin 2017 [72]	24 patients with hyperthyroidism, yet untreated and 24 voluntary normal persons	Multinodular goiter and Graves’ disease	Sclerostin	There was no difference in the serum sclerostin levels between the hyperthyroid group and the control group.
Mihaljević 2020 [70]	An amount of 30 patients with hypothyroidism, hyperthyroidism, and subclinical hyperthyroidism, as well as 10 euthyroid controls	Graves’ disease and subclinical hyperthyroidism due to thyroxine suppressive therapy	Sclerostin	Sclerostin levels were significantly elevated in hyperthyroidism compared to subclinical hyperthyroidism and control group
Mudri 2022 [32]	Longitudinal study included 37 patients in which serum sclerostin and DKK1 were measured at diagnosis of GD and after one year of ATD therapy	Graves’ disease	Sclerostin, DKK1	Significant decrease in serum sclerostin and significant increase in serum DKK1 in euthyroid state after one year

## 7. Conclusions

Due to the fact that hyperthyroidism can cause osteoporosis [74], there have been proposals to consider BMD as a standard test in patients with newly diagnosed hyperthyroidism to identify groups in need of further monitoring [75]. There are various studies in which hyperthyroidism led to a decrease in bone density [75,76,77,78]. Additionally, the risk of fracture is increased [79] in the first five years after the diagnosis of hyperthyroidism. Treatment with antithyroid drugs significantly reduced this risk independent of the dose [80]. However, an increase or normalization of bone density can occur even one to four years after hyperthyroidism is detected [4], and the increase in bone density can be partial, in the lumbar spine and hips, but with a decrease in the forearm [74,81]. Considering all the above stated, and the fact that bone density improves after antithyroid drug treatment, we assume that measuring bone density should not be mandatory in all patients with hyperthyroidism [82], but only in those that are already at risk for osteoporosis.

Sclerostin and DKK1 inhibit bone formation, promote bone resorption, and play an important role in maintaining bone balance. As a part of the Wnt pathway, they interact with thyroid hormones and their metabolism, and vice versa. As there are established treatments for osteoporosis using sclerostin inhibitor drugs, it would be interesting to further investigate their efficacy in hyperthyroidism for the reservation of bone mass. Moreover, additional research should be conducted on the interaction of the Wnt pathway and thyroid hormones in humans.

## References

[1] Chazenbalk G.D., Pichurin P., Chen C.R., Latrofa F., Johnstone A.P., McLachlan S.M., Rapoport B. (2002). Thyroid-stimulating autoantibodies in Graves disease preferentially recognize the free A subunit, not the thyrotropin holoreceptor. J. Clin. Investig..

[2] Antonelli A., Ferrari S.M., Ragusa F., Elia G., Paparo S.R., Ruffilli I., Patrizio A., Giusti C., Gonnella D., Cristaudo A. (2020). Graves’ disease: Epidemiology, genetic and environmental risk factors and viruses. Best Pract. Res. Clin. Endocrinol. Metab..

[3] Nicolaisen P., Obling M.L., Winther K.H., Hansen S., Hermann A.P., Hegedüs L., Bonnema S.J., Brix T.H. (2021). Consequences of Hyperthyroidism and Its Treatment for Bone Microarchitecture Assessed by High-Resolution Peripheral Quantitative Computed Tomography. Thyroid.

[4] Vestergaard P., Mosekilde L. (2003). Hyperthyroidism, bone mineral, and fracture risk--a meta-analysis. Thyroid.

[5] Lin C., Jiang X., Dai Z., Guo X., Weng T., Wang J., Li Y., Feng G., Gao X., He L. (2009). Sclerostin mediates bone response to mechanical unloading through antagonizing Wnt/beta-catenin signaling. J. Bone Miner. Res..

[6] Qiang Y.W., Barlogie B., Rudikoff S., Shaughnessy J.D. (2008). Dkk1-induced inhibition of Wnt signaling in osteoblast differentiation is an underlying mechanism of bone loss in multiple myeloma. Bone.

[7] Li J., Sarosi I., Cattley R.C., Pretorius J., Asuncion F., Grisanti M., Morony S., Adamu S., Geng Z., Qiu W. (2006). Dkk1-mediated inhibition of Wnt signaling in bone results in osteopenia. Bone.

[8] Delgado-Calle J., Sato A.Y., Bellido T. (2017). Role and mechanism of action of sclerostin in bone. Bone.

[9] Kim J.H., Liu X., Wang J., Chen X., Zhang H., Kim S.H., Cui J., Li R., Zhang W., Kong Y. (2013). Wnt signaling in bone formation and its therapeutic potential for bone diseases. Ther. Adv. Musculoskelet. Dis..

[10] Smith T.J., Hegedüs L. (2016). Graves’ Disease. N. Engl. J. Med..

[11] Bartalena L. (2013). Diagnosis and management of Graves disease: A global overview. Nat. Rev. Endocrinol..

[12] Chi H.C., Tsai C.Y., Tsai M.M., Yeh C.T., Lin K.H. (2019). Molecular functions and clinical impact of thyroid hormone-triggered autophagy in liver-related diseases. J. Biomed. Sci..

[13] Mancino G., Miro C., Di Cicco E., Dentice M. (2021). Thyroid hormone action in epidermal development and homeostasis and its implications in the pathophysiology of the skin. J. Endocrinol. Investig..

[14] Yen P.M. (2001). Physiological and molecular basis of thyroid hormone action. Physiol. Rev..

[15] Delitala A.P., Scuteri A., Doria C. (2020). Thyroid Hormone Diseases and Osteoporosis. J. Clin. Med..

[16] Welsh K.J., Soldin S.J. (2016). Diagnosis of endocrine disease: How reliable are free thyroid and total T3 hormone assays?. Eur. J. Endocrinol..

[17] Feldt-Rasmussen U., Krogh Rasmussen Å. (2007). Thyroid Hormone Transport and Actions. Pediatr. Adolesc. Med..

[18] Friesema E.C., Jansen J., Visser T.J. (2005). Thyroid hormone transporters. Biochem. Soc. Trans..

[19] Cardoso L.F., Maciel L.M., Paula F.J. (2014). The multiple effects of thyroid disorders on bone and mineral metabolism. Arq. Bras. Endocrinol. Metabol..

[20] Bassett J.H., Williams G.R. (2016). Role of Thyroid Hormones in Skeletal Development and Bone Maintenance. Endocr. Rev..

[21] Bianco A.C., Kim B.W. (2006). Deiodinases: Implications of the local control of thyroid hormone action. J. Clin. Investig..

[22] Cheng S.Y., Leonard J.L., Davis P.J. (2010). Molecular aspects of thyroid hormone actions. Endocr. Rev..

[23] Nicholls J.J., Brassill M.J., Williams G.R., Bassett J.H. (2012). The skeletal consequences of thyrotoxicosis. J. Endocrinol..

[24] Längericht J., Krämer I., Kahaly G.J. (2020). Glucocorticoids in Graves’ orbitopathy: Mechanisms of action and clinical application. Ther. Adv. Endocrinol. Metab..

[25] Chin Y.H., Ng C.H., Lee M.H., Koh J.W.H., Kiew J., Yang S.P., Sundar G., Khoo C.M. (2020). Prevalence of thyroid eye disease in Graves’ disease: A meta-analysis and systematic review. Clin. Endocrinol..

[26] Subekti I., Pramono L.A. (2018). Current Diagnosis and Management of Graves’ Disease. Acta Med. Indones.

[27] Burch H.B., Cooper D.S. (2015). Management of Graves Disease: A Review. JAMA.

[28] Zoričić-Cvek S.A., Bobinac D., Đudarić L., Cvijanović O. (2015). The remodeling of the skeleton. Med. Flumensis.

[29] Kenkre J.S., Bassett J. (2018). The bone remodelling cycle. Ann. Clin. Biochem..

[30] Williams G.R. (2013). Thyroid hormone actions in cartilage and bone. Eur. Thyroid J..

[31] Gorka J., Taylor-Gjevre R.M., Arnason T. (2013). Metabolic and clinical consequences of hyperthyroidism on bone density. Int. J. Endocrinol..

[32] Mudri D., Kizivat T., Mihaljević I., Ćurčić I.B. (2022). Wnt Inhibitors and Bone Mineral Density in Patients with Graves’ Disease Treated with Antithyroid Drugs: A Preliminary Prospective Study. Metabolites.

[33] Clevers H., Nusse R. (2012). Wnt/β-catenin signaling and disease. Cell.

[34] Baron R., Kneissel M. (2013). WNT signaling in bone homeostasis and disease: From human mutations to treatments. Nat. Med..

[35] Komiya Y., Habas R. (2008). Wnt signal transduction pathways. Organogenesis.

[36] Skah S., Uchuya-Castillo J., Sirakov M., Plateroti M. (2017). The thyroid hormone nuclear receptors and the Wnt/β-catenin pathway: An intriguing liaison. Dev. Biol..

[37] MacDonald B.T., Tamai K., He X. (2009). Wnt/beta-catenin signaling: Components, mechanisms, and diseases. Dev. Cell.

[38] Chen Y., Chen Z., Tang Y., Xiao Q. (2021). The involvement of noncanonical Wnt signaling in cancers. Biomed. Pharmacother..

[39] Lademann F., Tsourdi E., Hofbauer L.C., Rauner M. (2020). Thyroid Hormone Actions and Bone Remodeling—The Role of the Wnt Signaling Pathway. Exp. Clin. Endocrinol. Diabetes.

[40] Liu J., Xiao Q., Xiao J., Niu C., Li Y., Zhang X., Zhou Z., Shu G., Yin G. (2022). Wnt/β-catenin signalling: Function, biological mechanisms, and therapeutic opportunities. Signal Transduct. Target Ther..

[41] Flack J.E., Mieszczanek J., Novcic N., Bienz M. (2017). Wnt-Dependent Inactivation of the Groucho/TLE Co-repressor by the HECT E3 Ubiquitin Ligase Hyd/UBR5. Mol. Cell.

[42] Krishnan V., Bryant H.U., Macdougald O.A. (2006). Regulation of bone mass by Wnt signaling. J. Clin. Investig..

[43] Zheng J., Maerz W., Gergei I., Kleber M., Drechsler C., Wanner C., Brandenburg V., Reppe S., Gautvik K.M., Medina-Gomez C. (2019). Mendelian Randomization Analysis Reveals a Causal Influence of Circulating Sclerostin Levels on Bone Mineral Density and Fractures. J. Bone Miner. Res..

[44] Ke H.Z., Richards W.G., Li X., Ominsky M.S. (2012). Sclerostin and Dickkopf-1 as therapeutic targets in bone diseases. Endocr. Rev..

[45] Carrillo-López N., Martínez-Arias L., Fernández-Villabrille S., Ruiz-Torres M.P., Dusso A., Cannata-Andía J.B., Naves-Díaz M., Panizo S., On behalf of the European Renal Osteodystrophy (EUROD) Workgroup (2021). Role of the RANK/RANKL/OPG and Wnt/β-Catenin Systems in CKD Bone and Cardiovascular Disorders. Calcif. Tissue Int..

[46] Kubota T., Michigami T., Ozono K. (2009). Wnt signaling in bone metabolism. J. Bone Miner. Metab..

[47] Shan Y., Wang L., Li G., Shen G., Zhang P., Xu Y. (2019). Methylation of bone. Biochem. Cell Biol..

[48] Delgado-Calle J., Bellido T. (2015). Osteocytes and Skeletal Pathophysiology. Curr. Mol. Biol. Rep..

[49] Schaffler M.B., Kennedy O.D. (2012). Osteocyte signaling in bone. Curr. Osteoporos. Rep..

[50] Dallas S.L., Prideaux M., Bonewald L.F. (2013). The osteocyte: An endocrine cell… and more. Endocr. Rev..

[51] Sebastian A., Loots G.G. (2017). Transcriptional control of Sost in bone. Bone.

[52] Hu Y., Liu M., Xu S., Li S., Yang M., Su T., Yuan Z., Peng H. (2020). The Clinical Significance of Dickkopf Wnt Signaling Pathway Inhibitor Gene Family in Head and Neck Squamous Cell Carcinoma. Med. Sci. Monit..

[53] Huang Y., Liu L., Liu A. (2018). Dickkopf-1: Current knowledge and related diseases. Life Sci..

[54] Evenepoel P., D’Haese P., Brandenburg V. (2015). Sclerostin and DKK1: New players in renal bone and vascular disease. Kidney Int..

[55] Rachner T.D., Göbel A., Benad-Mehner P., Hofbauer L.C., Rauner M. (2014). Dickkopf-1 as a mediator and novel target in malignant bone disease. Cancer Lett..

[56] Paik J., Scott L.J. (2020). Romosozumab: A Review in Postmenopausal Osteoporosis. Drugs Aging.

[57] Dentice M., Marsili A., Zavacki A., Larsen P.R., Salvatore D. (2013). The deiodinases and the control of intracellular thyroid hormone signaling during cellular differentiation. Biochim. Biophys. Acta.

[58] Sirakov M., Skah S., Nadjar J., Plateroti M. (2013). Thyroid hormone’s action on progenitor/stem cell biology: New challenge for a classic hormone?. Biochim. Biophys. Acta.

[59] Natsume H., Sasaki S., Kitagawa M., Kashiwabara Y., Matsushita A., Nakano K., Nishiyama K., Nagayama K., Misawa H., Masuda H. (2003). Beta-catenin/Tcf-1-mediated transactivation of cyclin D1 promoter is negatively regulated by thyroid hormone. Biochem. Biophys. Res. Commun..

[60] Guigon C.J., Zhao L., Lu C., Willingham M.C., Cheng S.Y. (2008). Regulation of beta-catenin by a novel nongenomic action of thyroid hormone beta receptor. Mol. Cell Biol..

[61] Dentice M., Luongo C., Ambrosio R., Sibilio A., Casillo A., Iaccarino A., Troncone G., Fenzi G., Larsen P.R., Salvatore D. (2012). β-Catenin regulates deiodinase levels and thyroid hormone signaling in colon cancer cells. Gastroenterology.

[62] Ely K.A., Bischoff L.A., Weiss V.L. (2018). Wnt Signaling in Thyroid Homeostasis and Carcinogenesis. Genes.

[63] Wang L., Shao Y.Y., Ballock R.T. (2007). Thyroid hormone interacts with the Wnt/beta-catenin signaling pathway in the terminal differentiation of growth plate chondrocytes. J. Bone Miner. Res..

[64] Wang L., Shao Y.Y., Ballock R.T. (2009). Carboxypeptidase Z (CPZ) links thyroid hormone and Wnt signaling pathways in growth plate chondrocytes. J. Bone Miner. Res..

[65] Wang L., Shao Y.Y., Ballock R.T. (2010). Thyroid hormone-mediated growth and differentiation of growth plate chondrocytes involves IGF-1 modulation of beta-catenin signaling. J. Bone Miner. Res..

[66] Furuya F., Hanover J.A., Cheng S.Y. (2006). Activation of phosphatidylinositol 3-kinase signaling by a mutant thyroid hormone beta receptor. Proc. Natl. Acad. Sci. USA.

[67] O’Shea P.J., Kim D.W., Logan J.G., Davis S., Walker R.L., Meltzer P.S., Cheng S.Y., Williams G.R. (2012). Advanced bone formation in mice with a dominant-negative mutation in the thyroid hormone receptor β gene due to activation of Wnt/β-catenin protein signaling. J. Biol. Chem..

[68] Skowrońska-Jóźwiak E., Krawczyk-Rusiecka K., Lewandowski K.C., Adamczewski Z., Lewiński A. (2012). Successful treatment of thyrotoxicosis is accompanied by a decrease in serum sclerostin levels. Thyroid Res..

[69] Skowrońska-Jóźwiak E., Lewandowski K.C., Adamczewski Z., Krawczyk-Rusiecka K., Lewiński A. (2015). Mechanisms of Normalisation of Bone Metabolism during Recovery from Hyperthyroidism: Potential Role for Sclerostin and Parathyroid Hormone. Int. J. Endocrinol..

[70] Mihaljević O., Živančević-Simonović S., Lučić-Tomić A., Živković I., Minić R., Mijatović-Teodorović L., Jovanović Z., Anđelković M., Stanojević-Pirković M. (2020). The association of circulating sclerostin level with markers of bone metabolism in patients with thyroid dysfunction. J. Med. Biochem..

[71] Tsourdi E., Rijntjes E., Köhrle J., Hofbauer L.C., Rauner M. (2015). Hyperthyroidism and Hypothyroidism in Male Mice and Their Effects on Bone Mass, Bone Turnover, and the Wnt Inhibitors Sclerostin and Dickkopf-1. Endocrinology.

[72] Sarıtekin İ., Açıkgöz Ş., Bayraktaroğlu T., Kuzu F., Can M., Güven B., Mungan G., Büyükuysal Ç., Sarıkaya S. (2017). Sclerostin and bone metabolism markers in hyperthyroidism before treatment and interrelations between them. Acta Biochim. Pol..

[73] Tsourdi E., Colditz J., Lademann F., Rijntjes E., Köhrle J., Niehrs C., Hofbauer L.C., Rauner M. (2019). The Role of Dickkopf-1 in Thyroid Hormone-Induced Changes of Bone Remodeling in Male Mice. Endocrinology.

[74] Rozenberg S., Bruyère O., Bergmann P., Cavalier E., Gielen E., Goemaere S., Kaufman J.M., Lapauw B., Laurent M.R., De Schepper J. (2020). How to manage osteoporosis before the age of 50. Maturitas.

[75] Majima T., Komatsu Y., Doi K., Takagi C., Shigemoto M., Fukao A., Morimoto T., Corners J., Nakao K. (2006). Negative correlation between bone mineral density and TSH receptor antibodies in male patients with untreated Graves’ disease. Osteoporos. Int..

[76] Boonya-Ussadorn T., Punkaew B., Sriassawaamorn N. (2010). A comparative study of bone mineral density between premenopausal women with hyperthyroidism and healthy premenopausal women. J. Med. Assoc. Thai.

[77] Tuchendler D., Bolanowski M. (2013). Assessment of bone metabolism in premenopausal females with hyperthyroidism and hypothyroidism. Endokrynol. Pol..

[78] Barbosa A.P., Rui Mascarenhas M., Silva C.F., Távora I., Bicho M., do Carmo I., de Oliveira A.G. (2015). Prevalence of silent vertebral fractures detected by vertebral fracture assessment in young Portuguese men with hyperthyroidism. Eur. J. Endocrinol..

[79] Vestergaard P., Mosekilde L. (2002). Fractures in patients with hyperthyroidism and hypothyroidism: A nationwide follow-up study in 16,249 patients. Thyroid.

[80] Vestergaard P., Rejnmark L., Mosekilde L. (2005). Influence of hyper- and hypothyroidism, and the effects of treatment with antithyroid drugs and levothyroxine on fracture risk. Calcif. Tissue Int..

[81] Dhanwal D.K., Gupta N. (2011). Bone mineral density trends in Indian patients with hyperthyroidism—Effect of antithyroid therapy. J. Assoc. Physicians India.

[82] Deshmukh H., Papageorgiou M., Aye M., England J., Abdalla M., Sathyapalan T. (2021). Hyperthyroidism and bone mineral density: Dissecting the causal association with Mendelian randomization analysis. Clin. Endocrinol..

